# Experimental Growth Conditions affect Direct and Indirect Defences in two Cotton Species

**DOI:** 10.1007/s10886-023-01422-5

**Published:** 2023-05-09

**Authors:** Laura Chappuis, Alicia Egger, Gregory Roeder, Gaétan Glauser, Geoffrey Jaffuel, Betty Benrey, Luis Abdala-Roberts, Mary V. Clancy, Ted C. J. Turlings, Carlos Bustos-Segura

**Affiliations:** 1 FARCE laboratory, Institute of Biology, University of Neuchâtel, Rue Emile Argand 11, Neuchâtel, 2000 Switzerland; 2 Neuchâtel Platform of Analytical Chemistry, Institute of Chemistry, University of Neuchâtel, Avenue de Bellevaux 51, Neuchâtel, 2000 Switzerland; 3 Laboratory of Evolutionary Entomology, Institute of Biology, University of Neuchâtel, Rue Emile-Argand 11, Neuchâtel, 2000 Switzerland; 4 Departamento de Ecología Tropical, Campus de Ciencias Biológicas y Agropecuarias, Universidad Autónoma de Yucatán, Apartado Postal 4-116, Itzimná, Mérida, 97000 Yucatán Mexico

**Keywords:** Cotton, *Gossypium herbaceum*, *Gossypium hirsutum*, induced defence, chemical defence, herbivore-induced plant volatiles, parasitoids, terpenoids, growing conditions

## Abstract

Cotton has been used as a model plant to study direct and indirect plant defence against herbivorous insects. However, the plant growing conditions could have an important effect on the outcome of such plant defence studies. We examined how common experimental growth conditions influence constitutive and inducible defences in two species of cotton, *Gossypium hirsutum* and *G. herbaceum*. We induced plants by applying caterpillar regurgitant to mechanical wounds to compare the induction levels between plants of both species grown in greenhouse or phytotron conditions. For this we measured defence metabolites (gossypol and heliocides) and performance of *Spodoptera frugiperda* caterpillars on different leaves, the emission of plant volatiles, and their attractiveness to parasitic wasps. Induction increased the levels of defence metabolites, which in turn decreased the performance of *S. frugiperda* larvae. Constitutive and induced defence levels were the highest in plants grown in the phytotron (compared to greenhouse plants), *G. hirsutum* and young leaves. Defence induction was more pronounced in plants grown in the phytotron and in young leaves. Also, the differences between growing conditions were more evident for metabolites in the youngest leaves, indicating an interaction with plant ontogeny. The composition of emitted volatiles was different between plants from the two growth conditions, with greenhouse-grown plants showing more variation than phytotron-grown plants. Also, *G. hirsutum* released higher amounts of volatiles and attracted more parasitic wasps than *G. herbaceum*. Overall, these results highlight the importance of experimental abiotic factors in plant defence induction and ontogeny of defences. We therefore suggest careful consideration in selecting the appropriate experimental growing conditions for studies on plant defences.

**Supplementary Information:**

The online version contains supplementary material available at 10.1007/s10886-023-01422-5.

## Introduction

Plants have evolved a wide range of traits to defend themselves against herbivores (Karban and Baldwin 2007; Farmer 2014). These defensive traits can be direct, in the form of physical barriers (e.g. thorns and spines) or the production of toxic compounds (Bennett and Wallsgrove 1994; Agrawal et al. 1999), and can also be indirect, i.e. by attracting and sustaining natural enemies of herbivores (Dicke and Baldwin 2010; Schuman and Baldwin 2016; Turlings and Erb 2018). Plant defences can be constitutive or induced in response to herbivory, as is the case for secondary metabolites (Figueiredo et al. 2008). With regards to inducible defences, biotic stressors such as insect and pathogen attack are known to modify the expression of secondary metabolites as part of a strategy to resist the specific attacker (Karban & Baldwin, 2007; Tallamy & Raupp, 1991). Less well studied are the environmental conditions that affect expression of the genes and biosynthetic pathways involved in the production of defensive compounds, but it is evident that light conditions, temperature, nutrient status, and soil water content can strongly affect secondary metabolite production (Gouinguené and Turlings 2002; Ramakrishna and Ravishankar 2011).

Cotton plants are well suited for studying plant defence mechanisms as they employ distinct constitutive and inducible direct and indirect defences (Karban 1993; Loughrin et al. 1994; Röse et al. 1996, 1998; Opitz et al. 2008; Arce et al. 2021). The main direct chemical defence in several cotton species (*Gossypium* genus) is mediated by the lysigenous pigment glands that are present in all tissues, including the leaves (Longmore 1886; Opitz et al. 2008). These glands produce and store the sesquiterpenoid gossypol (Marchlewski 1899) and other related terpenoid aldehydes such as heliocides, which exhibit insecticidal activity (Adams et al. 1960; McAuslane et al. 1997; Stipanovic et al. 2006) and fungicidal properties (Puckhaber et al. 2002). These pigment glands also contain various volatile mono- and sesquiterpenoids (Loughrin et al. 1994; Opitz et al. 2008). The accumulation of these secondary metabolites is also inducible; the number of glands and their terpenoid content have been shown to increase following chewing herbivory by *Spodoptera exigua* (McAuslane et al. 1997; Bezemer et al. 2004; Opitz et al. 2008), but also in response to mere mechanical damage (Mamin et al., 2023). Another direct defence found in cotton is the presence of trichomes; Butler et al (1991) found that plants that had a dense covering of trichomes suffered lower damage from a variety of insects including leafhoppers and boll weevils, however the reverse is found for whiteflies, the density of which increased with trichome density (Butler et al. 1991).

Cotton also utilises several indirect methods of defence. The presence of extrafloral nectaries in cotton has been long documented (Fryxell 1979; Wäckers and Wunderlin 1999), and herbivory is known to increase the production of extra-floral nectar (Wäckers et al. 2001; Wäckers and Bezemer 2003), which is important for the attraction of herbivore enemies such as ants and parasitoids (Cook 1904; Heil 2015). Moreover, herbivore damage by the generalist *Helicoverpa zea* triggers the emission of volatile organic compounds (VOCs) that attract natural enemies (McCall et al. 1994). In addition to the immediate release of volatile compounds upon chewing herbivory (likely due to the rupture of pigment glands as leaf tissue is destroyed consequent to being consumed; (McCall et al. 1994)), cotton plants also *de novo* synthesise and release various additional VOCs a period of time after the initial attack, thereby adding to the signalling information of the volatile blend (Loughrin et al. 1994; Röse et al. 1996; Paré and Tumlinson 1997; Arce et al. 2021). This substantial and dynamic release of volatiles makes cotton an ideal plant for studies on the various functions of VOC emissions and their potential for application in pest control. As is the case for direct defence, indirect defence mechanisms within a species can be highly varied, and influenced by a number of factors. For instance, considerable quantitative and qualitative variation is observed in VOC profile emissions among different genotypes of the same species (Loughrin et al. 1995; Turlings et al. 1998; Halitschke et al. 2000; Degen et al. 2004; Clancy et al. 2016, 2023; Bustos-Segura and Foley 2018; Grof-Tisza et al. 2022). Although few studies have investigated the importance of environmental conditions on inducible volatile emissions, the growing conditions should have strong effects (Gouinguené and Turlings 2002; Olson et al. 2009). Given the key role abiotic environmental factors can play, we aimed to investigate whether the often-artificial conditions under which experimental cotton plants are grown influence induced plant defences.

The main objective of this study was to determine the extent to which environmental growth conditions impact the induction of direct and indirect chemical defences in cotton. To this end, we grew two cotton species, *Gossypium herbaceum* (Levant cotton) and *G. hirsutum* (upland cotton), in either a greenhouse or a phytotron. To look at induced defences, we mimicked herbivory by applying regurgitant from the larvae of the noctuid moth *Spodoptera frugiperda* to the wounds of mechanically damaged cotton leaves. *S. frugiperda* is a generalist that is not specifically adapted to feed on cotton, but it readily feeds on it and is one of the main pests of cotton (De Lange et al. 2020). Next, leaf concentrations of the terpenoid aldehyde gossypol and related heliocides were compared between damaged and undamaged plants. As we expected induction to increase the levels of terpenoid aldehydes mainly in younger tissues, we compared levels in the third and fourth leaves (second youngest and youngest leaves, respectively). We analysed the effects on herbivore performance by comparing *S. frugiperda* larval growth and survival on leaves from the differently treated plants. In addition, we measured the VOC emissions of damaged and undamaged plants, and using a six arms olfactometer we also measured the attractiveness of VOCs emitted by damaged plants to the parasitoid wasp *Cotesia marginiventris*.

## Materials and Methods

*Plants.* Seeds of *Gossypium herbaceum* (Samen Mauser, Switzerland) and *Gossypium hirsutum* (var. DP 147 RF, Agroscope, Switzerland) were planted individually in plastic pots (4 cm diameter, 11 cm high) in regular potting soil (Landi, Switzerland). Plants were grown in either a greenhouse or a phytotron. In summer, plants grown in the greenhouse were illuminated with natural light, while artificial light was supplied in autumn and winter. Phytotron conditions were set to 14 h of light per day (5000 lm∙m^− 2^, starting 08:00), temperature 25 °C, and 50% relative humidity. Plants were watered once a day with no addition of fertiliser. Plants were used in experiments at about 4–6 weeks post-germination, when the fourth leaf was beginning to emerge. All experiments were performed between September and December 2015.

*Insects.* Larvae of the fall armyworm (*Spodoptera frugiperda*, J.E. Smith, Lepidoptera: Noctuidae) were obtained from a colony maintained at the University of Neuchâtel, Switzerland (permit A192558). Larvae were reared on a beet army worm artificial diet (Frontier Scientific Services, DE, USA) under artificial light conditions and at ambient temperature (25 °C). The generalist parasitic wasp species *Cotesia marginiventris* (Cresson, Hymenoptera: Braconidae) was reared (permit A192632-1) as described in Tamò et al. (2006) by offering *Spodoptera littoralis* larvae (approximately three days old) to mated female wasps. The rearing colony originated from individuals obtained at the USDA-ARS, Biological Control and Mass Rearing Research Unit (Mississippi, USA). The rearing was occasionally replenished with individuals from field collections in Southern Mexico. Eggs of the African cotton leafworm *S. littoralis* were provided by Syngenta (Stein, Switzerland) and kept in an incubator. After emergence, larvae were placed on beet army worm artificial diet at room temperature.

*Collection of regurgitant. S. frugiperda* larvae (6th instar) were fed on *G. hirsutum* leaves one day before collection. Regurgitant collections were done in the morning. To induce regurgitation, we gently pinched the larval head region with two fingers (described in detail in Turlings et al., 1993). The regurgitant was collected with a 20 µl pipette and transferred to a 1.5 ml tube where it was kept on ice until its use shortly after.

### Direct Defence Experiments

*Induction of terpenoid aldehydes.* Leaf damage consisted of inflicting three wounds with a pair of forceps over a three-day period, each wound measuring approximately 5 × 20 mm. Immediately after mechanical damage, 5 µl of *S. frugiperda* regurgitant were applied to the upper side of each wound. On day one, the first true leaf was damaged; on days two and three, the second and third true leaves, respectively were damaged. Damage treatments took place between 09:00–10:00. Undamaged and mechanically damaged plants were kept under the same conditions within each environment, but physically separated (different chambers in the phytotrons and more than 4 m apart from each other in the greenhouse; the positions for each treatment were randomised for each set of plants).

*Terpenoid aldehydes extraction and analysis.* For each set of plants, we analyzed three undamaged and three induced plants grown in both growing conditions (phytotron and greenhouse) for both cotton species (n = 24). This experiment was replicated three times for a total of 72 plants. When the fourth leaf was fully developed and the fifth leaf was beginning to emerge, the third and fourth leaves counting from the oldest leaf (leaf 3 and leaf 4 hereafter) were harvested and immediately frozen in liquid nitrogen. Samples were stored at -80 °C until further use. Frozen leaves were ground into a fine powder under liquid nitrogen. 250 µl acetonitrile was added to ~ 50 mg of frozen powder along with 5 glass beads (1.25–1.65 mm diameter). The samples were homogenised for 3 min at 20 Hz using a beadmill (Retsch MM 300, Haan, Germany), then centrifuged for 5 min at 14,000 rpm. Recovered supernatant was centrifuged one more time, then 200 µl was transferred to a 2 ml glass vial for further analysis (Glauser et al. 2013). As gossypol is a highly unstable molecule, samples were prepared immediately before chemical analysis. Samples were analysed using ultra high-performance liquid chromatography (UHPLC) coupled to a UV/Vis detector (wavelength set to 288 nm). A 2.5 µl aliquot of each sample was injected onto a CORTECS UPLC C18 column (2.1 × 50 mm, 1.6 μm; Waters, Switzerland). The flow rate was held constant at 0.4 ml∙min^− 1^ and the temperature was kept at 25 °C. Mobile phase A consisted of 0.05% formic acid in water; mobile phase B consisted of 0.05% formic acid in acetonitrile. The following gradient was used: 30–90% mobile phase B in 6 min, 90–100% mobile phase B in 0.1 min, held at 100% for 2 min followed by re-equilibration at 30% mobile phase B for 0.1 min. Gossypol (Sigma-Aldrich, MO, USA) at known quantities was used as an external standard for quantification. Heliocides were tentatively identified by mass spectra profiles (NIST library).

*Herbivore performance.* In another set of plants, we measured the performance (growth and survival) of *S. frugiperda* larvae when feeding on *G. herbaceum* and *G. hirsutum* plants; for each species, 12 plants grown in greenhouse and 12 in phytotron conditions were used. At the third leaf stage, six plants of each group were induced as described above by mechanically damaging the leaves and applying caterpillar regurgitant, while the other six plants were left undamaged. Damaged and undamaged plants were grown under the same conditions within each environment, and kept physically separated until use when the fifth leaf started to develop.

When leaf 4 was completely developed (2–3 weeks after induction treatment), leaf 3 and 4 of each plant were excised and individually placed in Petri dishes (8.5 cm diameter) containing water-soaked filter paper. One larva was placed into each dish which was then sealed with parafilm, allowing it to feed on all parts of the leaf. Second instar *S. frugiperda* larvae were starved for 24 h before use. Only larvae weighing 1–3 mg were used. Larvae were weighed right before the experiment and 24 h after feeding. Larvae were fed for four days in total, and survival was checked every day. To maintain humidity inside the Petri dishes, the filter papers were re-humidified after 48 h. The experiment was conducted under natural light and at room temperature (25 °C) in a climatised laboratory. On the last day, larvae were removed. The leaves were then dried in an oven at 60 °C for two days and scanned to measure the consumed area using paint.net (ver. 4.0).

### Indirect Defence Experiments

*VOCs collection.* We collected volatiles from a total of 72 plants (the same plants used for the terpenoid aldehydes induction, but before leaf collection). Following the final induction (day three), the plants were carefully placed in a glass bottle (6 cm diameter, 32 cm high) for volatile collection (Turlings et al. 1993) and kept under artificial light. Volatiles were collected on filters containing 25 mg 80/100 Hayesep-Q adsorbent (Sigma, Switzerland) for two hours between 14:00–16:00 with a push-pull system at a rate of 900 ml∙min^− 1^ in and 800 ml∙min^− 1^ out (Turlings et al. 1998). Trapped volatiles were eluted with 100 µl dichloromethane. Two internal standards (200 ng n-octane and nonyl-acetate in 10 µl dichloromethane) were added to the eluent. Samples were then stored at -80 °C until analysis.

Gas chromatography – mass spectroscopy (GC-MS; Agilent 7890B-5977B) was used to analyse the samples. A 2 µl aliquot of each sample was auto-injected onto an Agilent HP-5MS column (30 m x 250 μm x 0.25 μm). Samples were analysed in pulsed splitless mode with helium at a constant rate of 0.9 ml∙min^− 1^. After injection, temperature was held at 40 °C for 3.5 min, increased to 100 °C at a rate of 8 °C∙min^− 1^ then to 230 °C at a rate of 5 °C∙min^− 1^ followed by a post run hold of 3 min at 250 °C. Compounds were identified by comparing their mass spectra with those from the NIST 05 spectral library, an in-house library, available commercial standards (Table S1) and Kovats retention index library (Lucero et al. 2009). Quantification of the compounds was based on the peak areas of the compounds relative to the peak areas of the internal standards (nonyl acetate) with a correction using a response factor. The response factor relative to nonyl acetate was calculated for the following standards: benzaldehyde, (Z)-3-hexenal, (E)-2-hexenal, (Z)-3-hexenol, (Z)-3-hexenyl acetate, nonanal, α-pinene, myrcene, β-ocimene, linalool, indole, α-copaene, β-caryophyllene, β-farnesene and α-humulene. For compounds with no available standard, we used the response factor of the standard of the same compound class closest in retention time. When there were differences in molecular mass between a given compound and its closest standard, the response factor was normalized based on the molecular mass (Kreuzwieser et al. 2014).

*Parasitoid attraction.* For the olfactometer assays the induction treatment was the same as described above, using a new set of plants (24 in total). Parasitoid attraction was tested in a six arm olfactometer set up as described by Turlings et al. (2004) in a room at 25 °C with artificial light. For each test, one induced plant of each cotton species (*G. herbaceum* and *G. hirsutum*) and of each growth conditions (greenhouse and phytotron) served as four odour sources, whereas the two remaining arms were left empty to serve as controls. On day three of induction, approximately 10 min after the last damage event, the four plants were each placed inside individual glass bottles (6 cm diameter, 32 cm high). Each bottle was connected to the central chamber of the olfactometer; the air flow through each arm was 1.1 L min^− 1^. In the early afternoon, a group of six naïve female *Cotesia marginiventris* parasitoid wasps were released in the central chamber. After 30 min, their choices were recorded, and the wasps were removed. Three groups of six wasps were tested with the same plants. This experiment was replicated on six different days with one set of plants per day. On the days prior to the experiments, female and male wasps were separated to prevent the females from being overly harassed.

*Statistical analysis.* The analyses were carried out using R version 4.1.3 (R Core Team, 2022). The concentrations of gossypol, heliocides H1, H2 and H3, the sum of all heliocides, and the consumed area by caterpillars and their mass gain were analysed with generalized linear models (GLM) using as explanatory factors: cotton species (*G. herbaceum versus G. hirsutum*), growth condition (greenhouse *versus* phytotron), induction status (undamaged *versus* damaged), and leaf stage (leaf 3 *versus* leaf 4). As the errors were not normally distributed we used GLMs with gamma distribution and a log link function in all cases. For model selection, we built models with no interactions, double, triple or up to quadruple interactions including all lower level interactions, then we compared the AIC (Aikaike’s information criterion) among the four models to select the model with lower AIC for each response variable. Whenever the top models showed a ∆AIC < 2, we selected the model with fewer parameters. A Wald’s chi square test (analysis of variance type II) was used to analyse the effects of each main factor and their interactions in the selected models.

*S. frugiperda* survival on the leaf 3 and leaf 4 were analysed separately. We applied a parametric survival regression model, fitting a Weibull distribution using the following fixed factors: cotton species (*G. herbaceum versus G. hirsutum*), growth condition (greenhouse *versus* phytotron), and induction status (undamaged *versus* damaged).

For VOC analysis, we selected the 27 most dominant compounds (Table S1). The overall differences between plant treatments in VOC composition was tested with a redundancy analysis (RDA), using the normalised matrix of all compounds as the response variable, and growing conditions, species, and induction status as the explanatory variables, including all the double interactions. Total release of VOCs, green leaf volatiles, monoterpenes, sesquiterpenes, and homoterpenes were categorised. To compare the amounts of total volatiles (expressed in ng) among treatments, data were analysed with a Gamma GLM and a log link with the factors cotton species, growing conditions and induction status. The model selection approach was followed as above for direct defence and leaf consumption.

Individual compounds and the sum of each VOC category were analysed with the same GLM procedure, but using only models with all main factors and double interactions, which was the best model in most of the previous analyses. A false discovery rate correction was applied to the P values of each factor to account for the multiple comparisons. Results from the parasitoid attraction tests were analysed with a GLM using a Poisson distribution, with number of wasps choosing an arm in each assay as response variable and arm treatment as the explanatory variable. A model including the set of plants as a random factor was significantly worse than the fixed effects model and their results showed no substantial differences. The multiple comparisons among treatments were obtained by performing a Tukey test with the R package “emmeans” (Lenth et al. 2018).

## Results

**Fig. 1:**
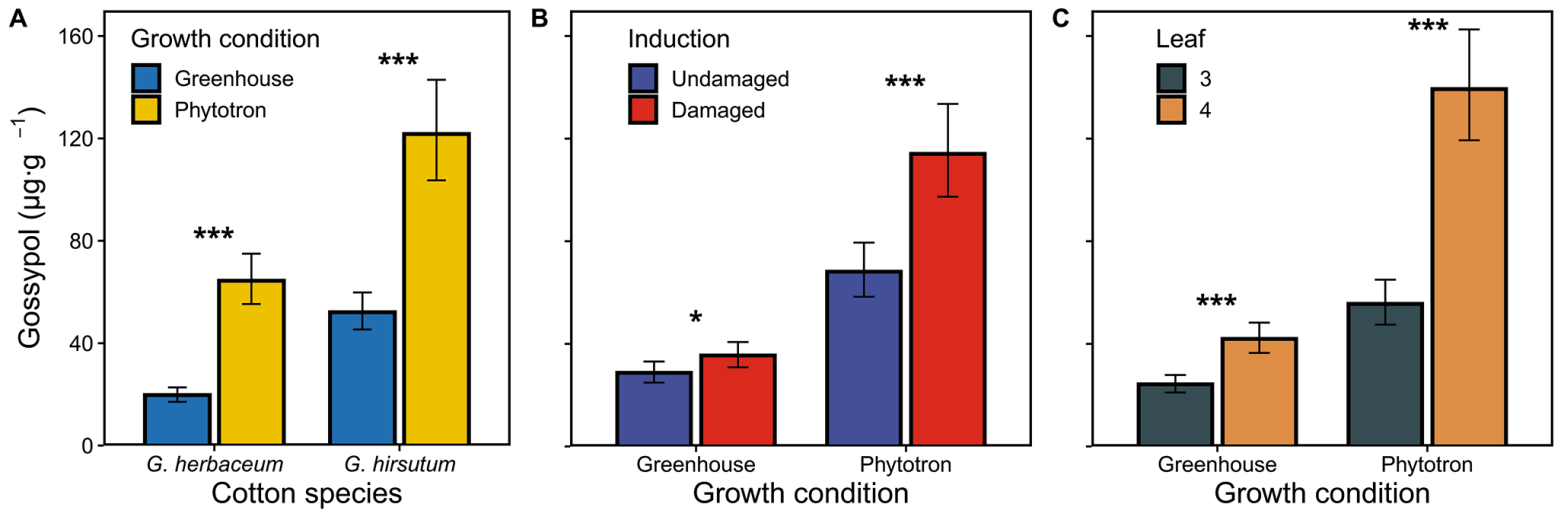
Effects of species and experimental conditions on gossypol concentrations in leaves. **A**: cotton species (*G. herbaceum* or *G. hirsutum*) by growth condition (greenhouse or phytotron); **B**: growth condition by induction status (undamaged or damaged); and **C**: growth condition by leaf stage (leaf 3 or leaf 4). Values for each panel are marginal mean estimates from the GLM for focal factors at averaged levels of non-focal factors. Error bars indicate confidence intervals (95%). Undamaged = plants kept intact as control, damaged = plants induced by mechanical damage and application of caterpillar regurgitant. Probability levels: * P < 0.05, ** P < 0.01, *** P < 0.001

*Induction of terpenoid aldehydes.* All the main factors (cotton species, growth condition, induction status, and leaf stage) significantly influenced gossypol leaf concentration, but also the interactions of growth condition by species, induction by growth condition and leaf stage by growth condition (Table 1). *G. hirsutum* contained on average 127% more gossypol than *G. herbaceum* plants (Fig. 1A). Plants grown in the phytotron produced considerably more gossypol than plants grown in the greenhouse. This difference was higher in *G. herbaceum* (230%) than in *G. hirsutum* (130%) (Fig. 1A). Moreover, damaged plants produced more gossypol than undamaged plants, but this difference was higher in the phytotron (70%) than in the greenhouse (20%) (Fig. 1B). Leaf 4 had more gossypol than leaf 3, with a greater difference between plants grown under phytotron (150%) than under greenhouse conditions (70%) (Fig. 1D). Other interactions were not statistically significant (Table 1).

**Table 1:** Summary of the Anova type II Wald’s chi square tables for models analysing variation in cotton and insect traits. The models included the factors Species (*G. herbaceum* vs. *G. hirsutum*), growing Condition (phytotron vs. greenhouse), Induction (undamaged vs. damaged) and Leaf stage (leaf 3 vs. leaf 4) and the interactions were selected according to the lowest AIC. The χ^2^ values for each factor are shown, with bold text for significant values (α = 0.05). The effect of the interaction (below the statistical parameter) describes the direction of the interaction (in which level the effect of another factor was higher). Df = 1 for all the factors. Asterisks indicate the level of significance: *(P < 0.05), **(P < 0.01), ***(P < 0.001). Abbreviations - her: *G. herbaceum*; hir: *G. hirsutum*; phy: phytotron. ∆AIC values indicate the increment in AIC from the selected model to the next best model. When ∆AIC < 2, the model with fewer parameters was selected

Trait	∆AIC	Species (S)	Condition (C)	Induction (I)	Leaf (L)	S*C	S*I	S*L	C*I	C*L	L*I
Gossypol	2.72	116.52***	179.08***	21.18***	88.69***	4.97* **C + in her**	1.15	1.05	4.11* **I + in phy**	6.02* **L + in phy**	1.75
Heliocide H1	0.57	101.06***	61.9***	50.15***	49.67***	0.16	1.30	7.66** **L + in hir**	14.88*** **I + in phy**	2.92	8.34** **I + in leaf4**
Heliocide H2	2.52	64.35***	29.76***	26.01***	25.50***	0.01	0.52	2.95	9.34** **I + in phy**	6.88** **L + in phy**	3.92* **I + in leaf4**
Heliocide H3	0.36	1.75	0.05	0.31	1.37	-	**-**	-	-	-	**-**
Total heliocides	5.61	26.94***	9.3**	5.73*	9.17**	0.16	1.63	0.45	2.63	6.60* **L + in phy**	3.07
Leaf consumption	2.58	5.54*	27.12***	28.40***	9.23**	0.09	3.99* **I + in her**	0.78	0.46	8.92** **L + in phy**	3.98* **I + in leaf4**
Mass gain	3.69	7.66**	20.83***	2.28	1.07	-	-	-	**-**	-	-
Total volatiles	-0.84	10.21**	0.49	314.45***	-	3.00	6.55* **I + in hir**	-	11.06*** **I + in phy**	-	-

**Fig. 2:**
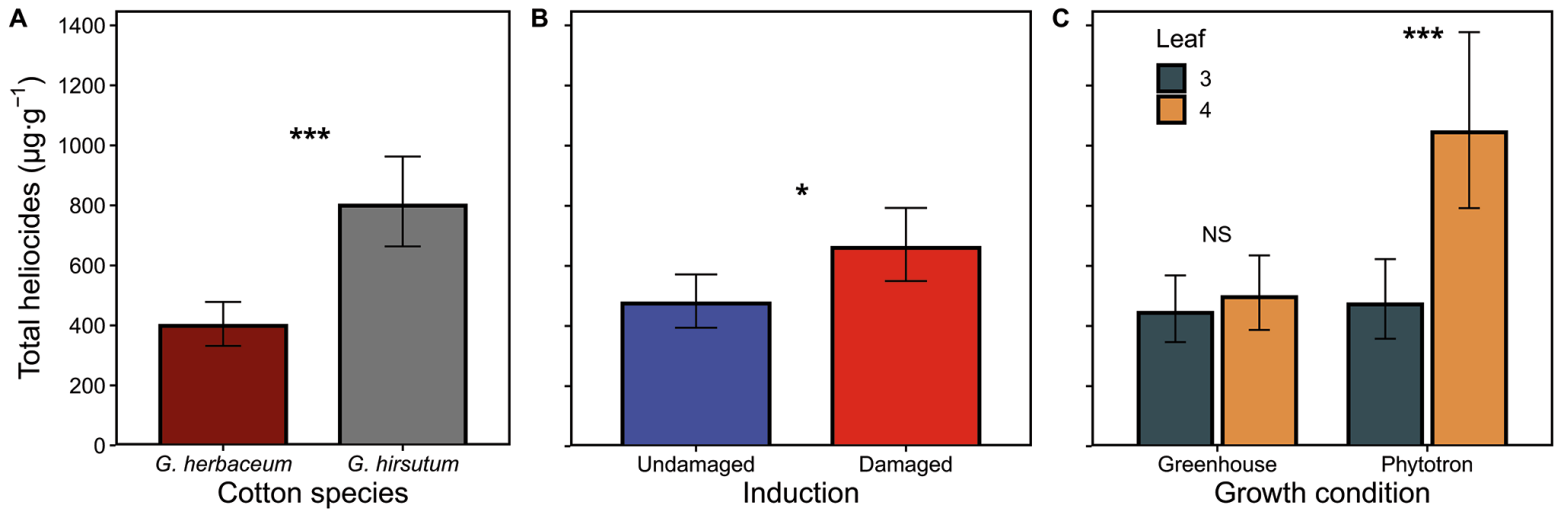
Effects of species and experimental conditions on the sum of three heliocides concentrations in leaves. **A**: cotton species (*G. herbaceum or G. hirsutum*); **B**: induction status (undamaged or damaged), and **C**: the interaction between growth condition (phytotron or greenhouse) and leaf stage (leaf 3 or leaf 4). Values for each panel are marginal mean estimates from the GLM for focal factors at averaged levels of non-focal factors. Error bars indicate confidence intervals (95%). Undamaged = plants kept intact as control, damaged = plants induced by mechanical damage and caterpillar regurgitant applications. Probability levels: * P < 0.05, ** P < 0.01, *** P < 0.001, NS: P > 0.05

When taking the three heliocides together, their concentration was significantly affected by all the main factors, plus the interaction of growth condition by leaf stage (Table 1). Heliocides concentration in *G. hirsutum* was two times the concentration measured in *G. herbaceum* (Fig. 2A). Damaged plants contained 40% more heliocides than the undamaged plants (Fig. 2B). The leaf stage interacted with growth conditions, as heliocides concentration was 120% higher in leaf 4 compared to leaf 3 in phytotron conditions, but they were not statistically different in the greenhouse (Fig. 2C). In other words, the difference between growth conditions was observed only in leaf 4 but not in leaf 3. Other interactions were not statistically significant (Table 1).

Heliocide H1 and heliocide H2 concentrations followed a similar pattern to that observed for the summed heliocides (Fig. S1 and S2). All main factors including growth condition, cotton species, induction status, and leaf stage significantly influenced heliocides H1 and H2 concentrations; however, other interactions were also significant (Table 1). In *G. herbaceum*, the difference in concentration of heliocide H1 between leaf 4 and leaf 3 was higher than in *G. hirsutum*. Plants grown in the phytotron produced more heliocides H1 and H2 in response to damage than plants grown in the greenhouse. The difference in heliocide H2 concentrations between leaf 3 and leaf 4 was higher in phytotron plants than in greenhouse plants. The induction in response to damage of heliocides H1 and H2 were higher in leaf 4 than in leaf 3. Contrarily, none of the main factors influenced the concentration of heliocide H2 (Fig. S3; Table 1).

**Fig. 3:**
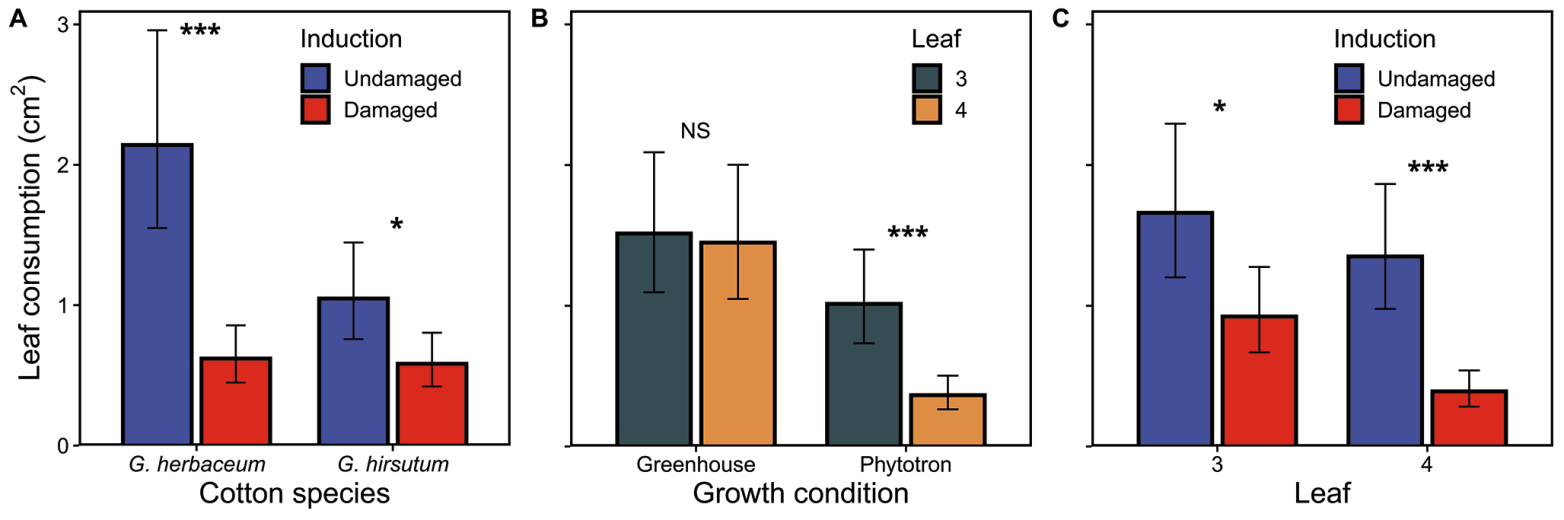
Effects of species and experimental conditions on leaf area consumed by *S. frugiperda *during 5 consecutive days. **A**: cotton species (*G. herbaceum* or *G. hirsutum*) by induction status (undamaged or damaged); **B**: growth condition (phytotron or greenhouse) by leaf stage (leaf 3 or leaf 4); **C**: leaf stage by induction status. Values for each panel are marginal mean estimates from the GLM for focal factors at averaged levels of non-focal factors. Error bars indicate confidence intervals (95%). Undamaged = plants kept intact as control, damaged = plants induced by mechanical damage and caterpillar regurgitant application. Probability levels: * P < 0.05, ** P < 0.01, *** P < 0.001, NS: P > 0.05

*Consumed leaf area.* All the main factors influenced the leaf consumption by caterpillars. The interactions species by induction status, growth condition by leaf stage, and leaf stage by induction status also significantly influenced leaf consumption by *S. frugiperda* caterpillars (Table 1). Consumption was 50% higher on *G. herbaceum* than on *G. hirsutum* leaves. Caterpillars ate 250% more on undamaged than on damaged *G. herbaceum* plants, but only 80% more on *G. hirsutum* plants (Fig. 3A). Leaf 3 was more consumed than leaf 4 (180%) for plants grown in the phytotron, but this difference was not significant for plants grown in the greenhouse (Fig. 3B). In addition, the difference in consumption between damaged and undamaged plants was higher in leaf 4 (250%) than in leaf 3 (80%) (Fig. 3C).

**Fig. 4:**
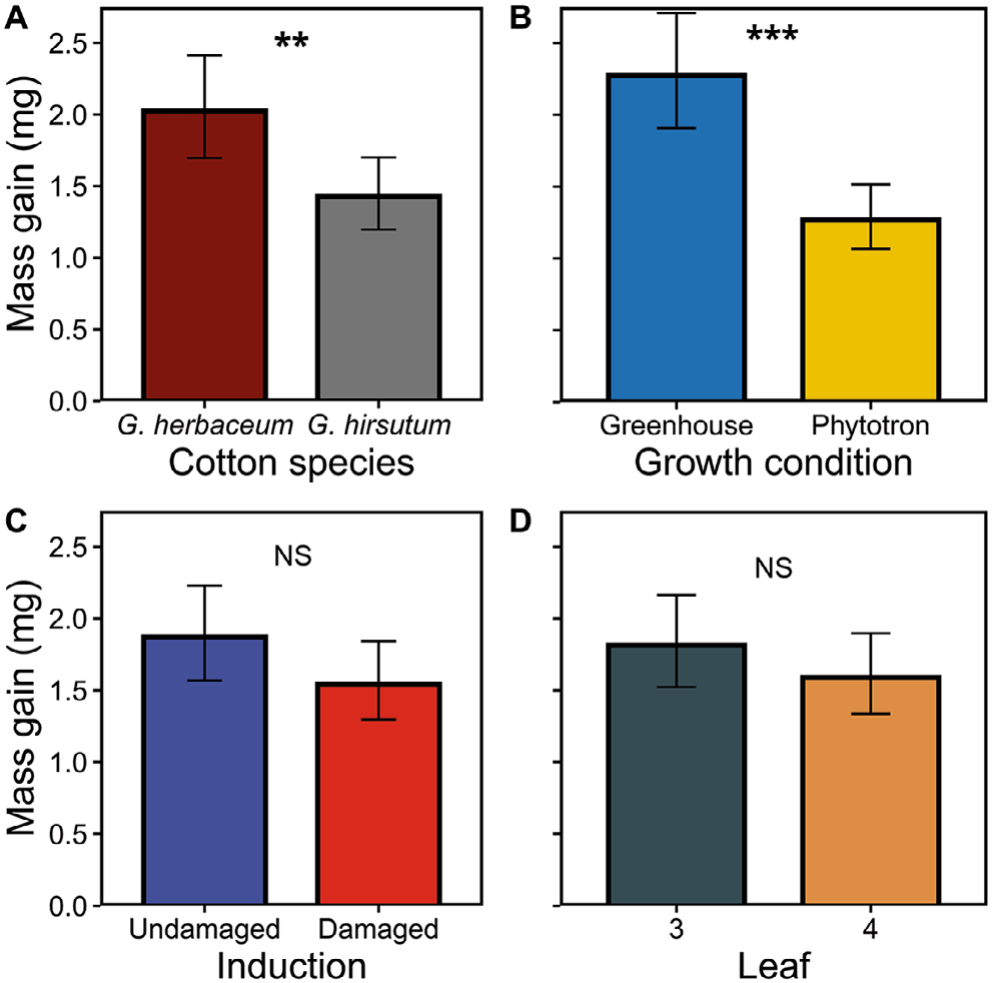
Effects of species and experimental conditions on mass gain (mg) by*S. frugiperda*larvae after 24 h of feeding. **A**: cotton species (*G. herbaceum* or *G. hirsutum*); **B**: growth condition (phytotron or greenhouse); **C**: induction status (undamaged or damaged); and **D**: leaf stage. Values for each panel are marginal mean estimates from the GLM for focal factors at averaged levels of non-focal factors. Error bars indicate confidence intervals (95%). Undamaged = plants kept intact as control, damaged = plants induced by mechanical damage and caterpillar regurgitant application. Units represent the number of pixels that covered the surface eaten by caterpillars. Probability levels: * P < 0.05, ** P < 0.01, *** P < 0.001, NS: P > 0.05

*Caterpillar mass gain. S. frugiperda* caterpillars gained significantly more mass (41%) in 24 h on *G. herbaceaum* than on *G. hirsutum* (Fig. 4A; Table 1). Caterpillars fed on leaves from greenhouse plants gained 79% more mass than on leaves from phytotron plants (Fig. 4B). On the other hand, induction status and leaf stage did not significantly influence the caterpillar mass gain (Fig. 4C and D; Table 1).

*Caterpillar survival.* For leaf 3, caterpillars survived better on plants grown in the greenhouse than in the phytotron (χ^2^_(1)_ = 4.86, P = 0.028), but survival was not affected by either cotton species or induction status (both P > 0.85). The survival of caterpillars that were fed on leaf 4 was also affected by growing condition (χ^2^_(1)_ = 21.73, P < 0.0001). Induction significantly reduced survival of *S. frugiperda* caterpillars on leaf 4 (χ^2^_(1)_ = 11.65, P < 0.0001). Finally, there was no significant difference in survival on leaf 4 between the two cotton species (χ^2^_(1)_ = 0.42, P = 0.52).

**Fig. 5:**
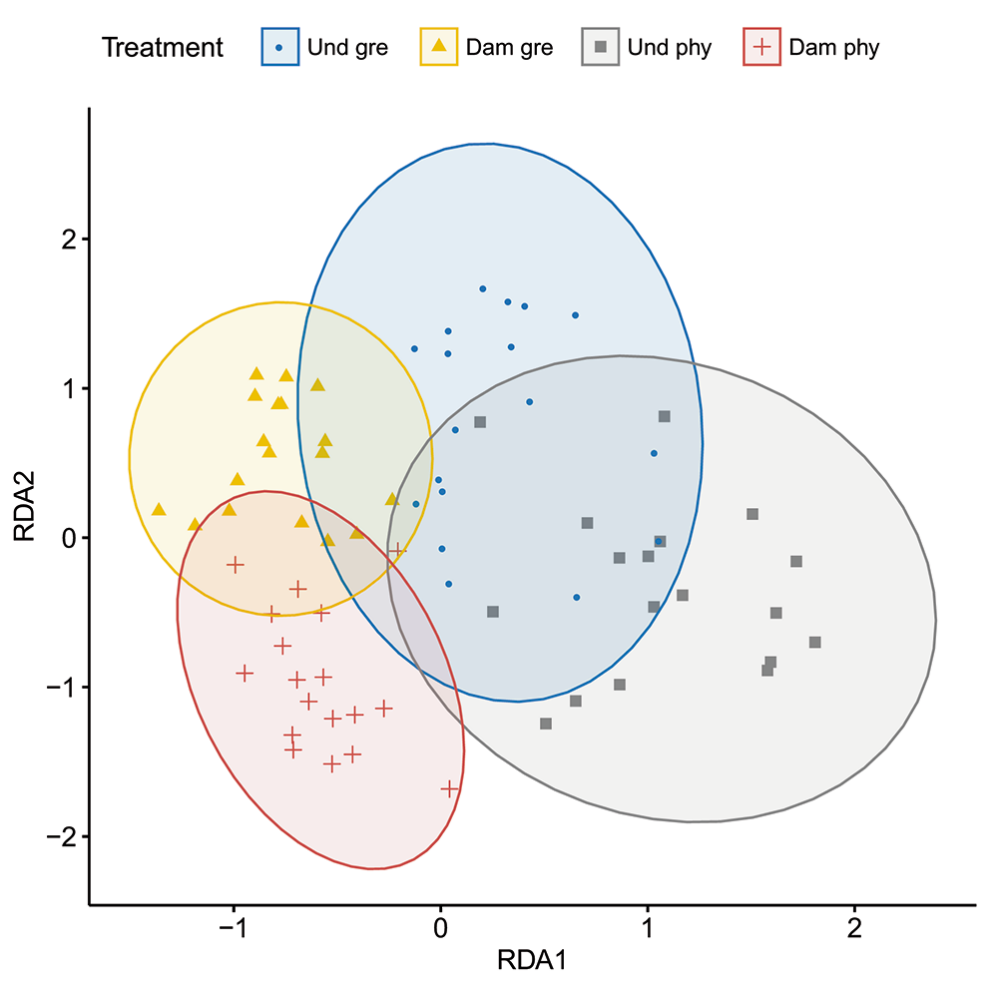
Results of a redundancy analysis (RDA) of the emitted VOCs by cotton plants under different conditions. Each dot represents an individual plant. Four treatments are represented: undamaged plants grown in the greenhouse (“Und gre”, blue circles) or in the phytotron (“Und phy”, purple squares), and damaged plants grown in the greenhouse (“Dam gre”, yellow triangles) or in the phytotron (“Dam phy”, red crosses). Each semi-transparent circle represents the confidence interval (95%) of each treatment group

*VOC collection.* The RDA analysis showed a difference in the volatile composition between damaged and undamaged plants (χ^2^_(1)_ = 258.9, P < 0.0001) and between growing conditions (χ^2^_(1)_ = 33.46, P < 0.0001). In addition there was an interaction between the damage treatment and the growing location (χ^2^_(1)_ = 12.22, P = 0.0005), with damaged plants showing more significant differences between growing locations than undamaged plants (Fig. 5). Damaged plants grown in the phytotron emitted more limonene, (E)-2-hexenal, (E)-β-ocimene, 1-decyne than greenhouse-grown plants (Fig. S4). The main effects of cotton species and the other interactions on volatile composition were not statistically significant.

**Fig. 6:**
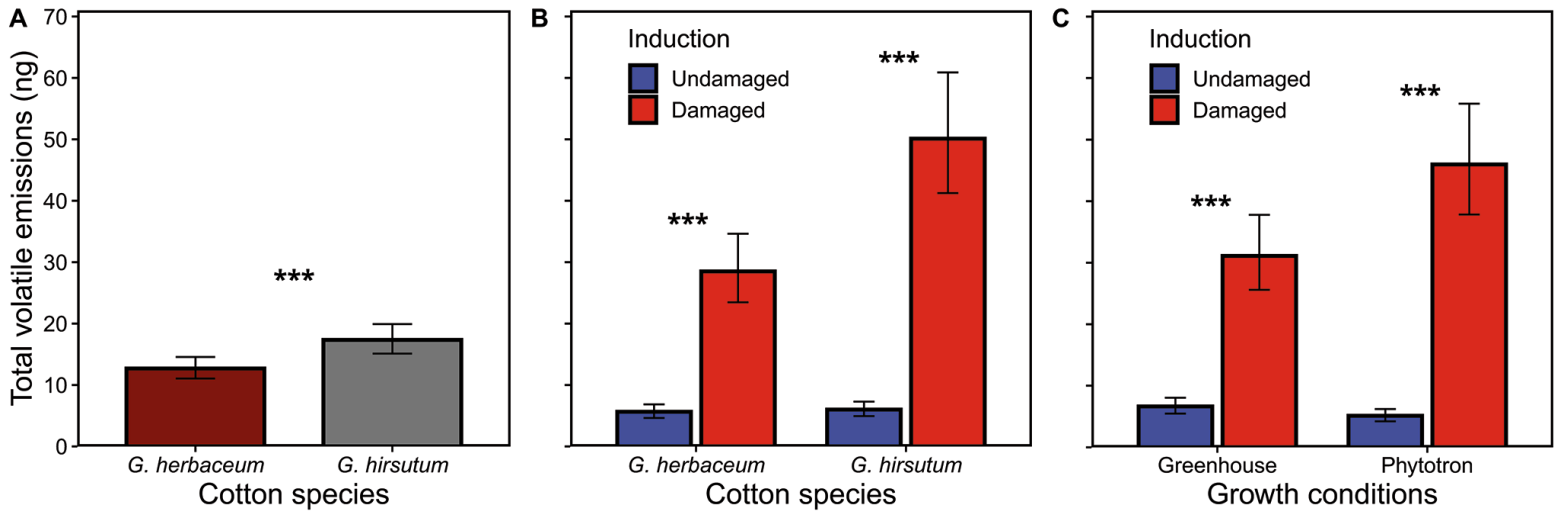
Effects of species and experimental conditions on total emissions (ng) of volatile organic compounds per plant. **A**: cotton species (*G. herbaceum* or *G. hirsutum*); and **B**: growing conditions (phytotron or greenhouse) by induction status (undamaged or damaged). The volatile collections were carried out over 2 h. Values for each panel are marginal mean estimates from the GLM for focal factors at averaged levels of non-focal factors. Error bars indicate confidence intervals (95%). Undamaged = plants kept intact as control, damaged = plants induced by mechanical damage and caterpillar regurgitant application. Probability levels: * P < 0.05, ** P < 0.01, *** P < 0.001, NS: P > 0.05

Overall, species and induction status affected the amount of total emitted volatiles. The interactions of induction by species and induction by growing conditions were statistically significant (Table 1). The emission of total volatiles by *G. hirsutum* plants was 37% higher than by *G. herbaceum* (Fig. 6A). When comparing VOC induction between *G. herbaceum* and *G. hirsutum*, damaged plants emitted 405% and 733% more volatiles than undamaged plants, respectively. When plants were grown in the greenhouse, induction increased VOC emissions by 368%, whereas this increment was 799% in plants grown in the phytotrons (Fig. 6B). In other words, undamaged plants grown in greenhouse emitted more volatiles than undamaged plants grown in the phytotron, but this effect was reversed for damaged plants.

For the sum of green leaf volatiles (GLVs), damaged plants released significantly more GLVs than undamaged plants (P < 0.001, Table S1). After a correction for the false discovery rate, other main factors and interactions were not statistically significant.

Of the identified terpenes, monoterpenes were the most abundant followed by sesquiterpenes and homoterpenes. The sum of each terpene category was significantly higher in induced than in undamaged plants (Table S1). Monoterpenes were affected by the interaction between induction status and species, and induction and growing condition. Monoterpenes were more induced by damage in *G. hirsutum* than in *G. herbaceum*, and their emissions were higher by phytotron grown plants compared to greenhouse grown plants (P < 0.001, Table S1). The emissions of homoterpenes were also higher for phytotron plants; however, this effect of induction was reversed for sesquiterpene emissions (P < 0.001). In addition, sesquiterpene emissions were higher for *G. hirsutum* than for *G. herbaceum* (P = 0.029).

The inducible aromatic compound indole was significantly affected by an interaction between growth condition and induction status (P < 0.001, Table S1). Plants grown in the phytotron released more indole when damaged than plants grown in the greenhouse.

**Fig. 7:**
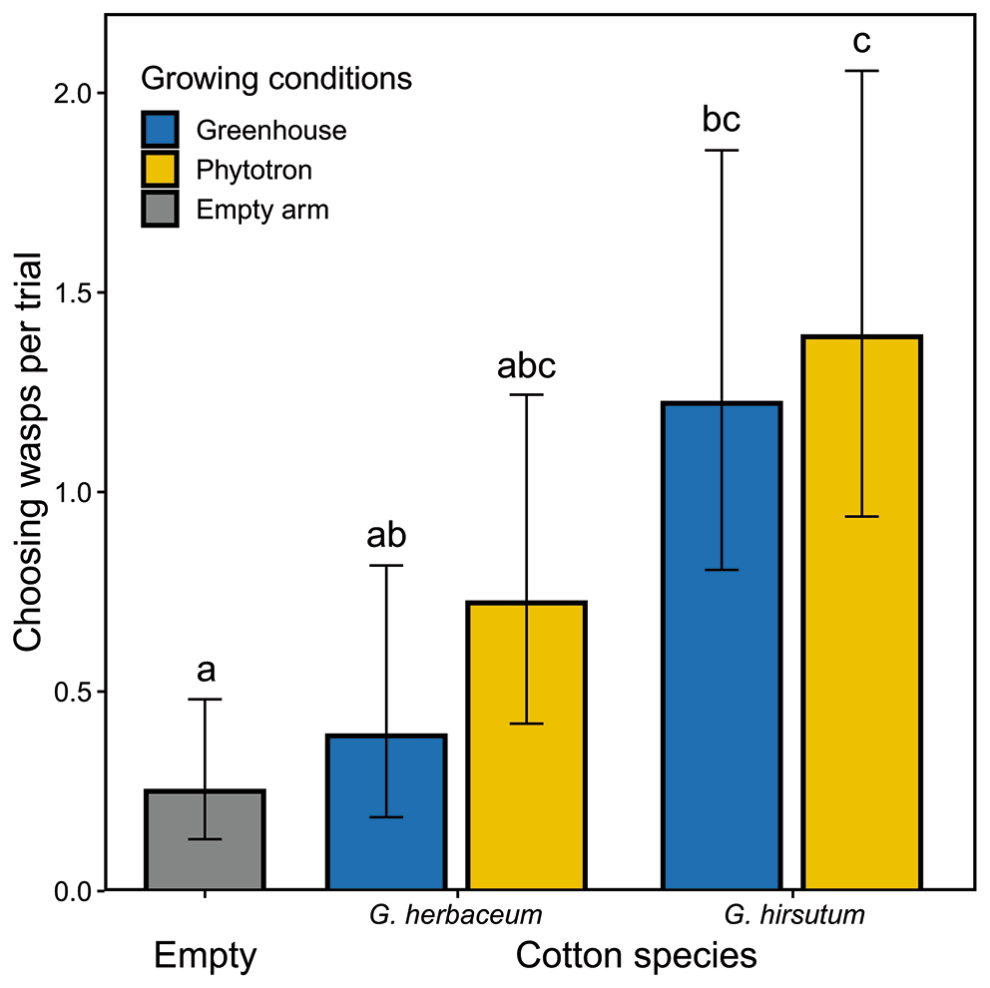
Number of *Cotesia marginiventris* females attracted to different cotton odour sources in a 6 arm-olfactometer. The odours were produced by mechanically damaged and regurgitant treated *G. herbaceum* and *G. hirsutum* plants grown in a phytotron or a greenhouse. For each trial six naive female wasps were released in the olfactometer arena. Values are the marginal mean estimates from the GLM. Error bars indicate confidence intervals (95%). Empty = empty odour vessels (mean value for the two empty vessels per trial). Different letters indicate significant differences between treatments (α = 0.05)

*Parasitoid attraction.* Approximately 70% of the female parasitoids chose an arm in the olfactometer (76 out of 108 wasps). The five olfactometer treatments (empty bottle, induced plants of *G. herbaceum* or *G. hirsutum*, grown in phytotron or greenhouse conditions) were differentially attractive to the wasps (χ_(4)_ = 32, P < 0.001; Fig. 7). The volatiles from induced *G. hirsutum* plants were significantly more attractive to wasps than clear air regardless of the growing conditions (phytotron: P < 0.001; greenhouse: P < 0.001), while induced *G. herbaceum* showed a comparatively low attractiveness (Fig. 7). There was a marginally significant difference in attractiveness between *G. herbaceum* and *G. hirsutum* plants grown in the greenhouse (P = 0.06), with wasps preferring *G. hirsutum*.

## Discussion

The purpose of this study was to investigate the effects of plant growth environments on the direct and indirect chemical defences in *Gossypium hirsutum* and *Gossypium herbaceum*. These two cotton species originate from Mesoamerica and sub-Saharan Africa/Arabia, respectively (Wendel et al. 2010). We compared plants that were grown in a greenhouse or phytotron, and that were undamaged or damaged by mechanically wounding the leaves and applying caterpillar regurgitant to injured areas. The induction treatment triggered an increase in gossypol and heliocide levels in both species. The inducibility of these terpenoid aldehydes is known for both *G. hirsutum* (Agrell et al. 2004; McAuslane et al. 1997) and *G. herbaceum* (Bezemer et al. 2004) and is shown here to be affected by the growing conditions. For both species, this increase in the concentration of chemical defence compounds upon induction resulted in *Spodoptera frugiperda* caterpillars consuming less leaf tissue and exhibiting higher mortality on the youngest leaves of induced plants. However, their mass gain after 24 h was only marginally affected by induction; 24 h of feeding might not be sufficient to observe the effects on their growth rate. The two cotton species also differed in inducible VOC emissions, with these differences affecting parasitic wasp attraction.

### Direct Chemical Defences

*G. hirsutum* leaves contained on average almost twice the concentration of terpenoid aldehydes as the leaves of *G. herbaceum*. *S. frugiperda* caterpillars fed more and gained more mass on *G. herbaceum* than on *G. hirsutum*, indicating that the performance of the caterpillars was related to concentration of these chemical defences. That the caterpillars performed worse on plants that contained more terpenoid aldehydes was also evident from the comparisons between growing conditions. Phytotron grown plants contained higher levels of terpenoid aldehydes compared to plants grown in the greenhouse. This might be explained by the fact that plants grown in the phytotron were subject to higher humidity and artificial light, and abiotic stress is known to increase gossypol levels in cotton plants (Wang et al. 2015). We also found that growing conditions had an impact on the differences in terpenoid aldehydes content between leaf 3 and leaf 4. Overall, terpenoid aldehyde levels in the youngest leaf (leaf 4) were higher in plants grown in the phytotron than in plants grown in the greenhouse. Cotton plants grown in the greenhouse took longer for their fourth leaf to develop (approximately 4 weeks compared to 3 weeks for plants grown in the phytotron). Eisenring et al. (2017), found gossypol concentration to decline when leaves were collected 14 days after an induction event compared to collection right after or seven days after induction. Heliocides, however, were highly induced regardless the time of leaf collection. In our study, gossypol and heliocides were less induced in greenhouse than in phytotron conditions, ruling out an effect of the time at leaf collection. This indicates that, as the new leaves finished developing after the induction treatment, the differential concentrations of defence compounds in these leaves may be linked to differences in investment to protect newly grown leaves under different abiotic conditions.

The defence compound contents corresponded perfectly with how plant growth conditions affected leaf consumption and mass gain of larvae after 24 h of feeding. Overall, larvae ate more and gained more mass when feeding on plants grown in the greenhouse than on plants grown in the phytotron, with a more pronounced effect when feeding on leaf 4. Larval survival was also higher after five days when feeding on plants grown in the greenhouse. This indicated that the plants grown in the phytotron were more resistant, as they contained more terpenoid aldehydes and responded stronger to damage, which affected herbivore performance.

The effect we observed of leaf stage on terpenoid aldehyde concentrations is in line with what has been reported in previous studies (Bezemer et al. 2004; McAuslane et al. 1997; McAuslane and Alborn 1998). We found that younger leaves contained higher levels of terpenoid aldehydes than older leaves. This result is consistent with the optimal-defence theory, which predicts that plants invest more in defensive compounds in parts with higher value for fitness, such as young leaves (McKey 1979; Ohnmeiss and Baldwin 2000; Rhoades 1979). Larvae consumed more leaf tissue when feeding on older leaves in comparison to younger leaves from plants grown in the phytotron, implying that herbivores feed more on the less defended older leaves. Larval survival was negatively influenced by the induction of defences, but only when feeding on young leaves. This was not surprising as induction had a more pronounced effect on terpenoid aldehyde levels in older leaves than in younger leaves, in accordance with a higher investment in the defence of younger tissue. The difference in defensive compounds between leaf stages was also more pronounced in phytotron-grown plants, which indicated that growth conditions have an influence on defence ontogeny. In accordance with previous studies, we found that the increase of the heliocide H1 after damage was greater than for the heliocides H2 and H3 (Agrell et al. 2004; Bezemer et al. 2004; McAuslane et al. 1997; McAuslane and Alborn 1998), which could also result in differences in the effect of heliocides on antiherbivory defences. Overall, our results confirm the effectiveness of cotton direct defences against *S. frugiperda* larvae by showing that high levels of terpenoid aldehydes in leaf tissues is associated with an overall negative effect on larval performance. Caterpillars fed less and died more on plants with higher levels of these defensive compounds. In concurrence with previous studies, we can conclude that gossypol and heliocides are feeding deterrents and are toxic to herbivores (McAuslane et al. 1997; McAuslane and Alborn 1998).

### Indirect Chemical Defences

Also, as expected from previous studies (Loughrin et al. 1994, 1995; Turlings et al. 1993), we found that mechanically damaging leaves and applying caterpillar regurgitant to the wounds induced a considerable increase in VOC emissions. VOC emissions in response to induction also different depending on species; we found that *G. hirsutum* released higher amounts than *G. herbaceum*. The induction of VOC emissions was found to be also influenced by growth conditions; plants grown in the phytotron released lower amounts of constitutive volatiles but higher amounts of herbivore-induced plant volatiles (HIPVs) than greenhouse grown plants. The HIPV composition (volatile profiles) also varied depending on whether the plants were grown in the greenhouse or phytotron. These findings imply that indirect defence can be affected both quantitatively and qualitatively by biotic as well as abiotic factors. Interestingly, both species exhibited similar VOC compositions.

The attraction of naive female parasitic wasps (*Cotesia marginiventris*) to the cotton plants was consistent with the results found for the total release of VOCs. The highest amounts were released by *G. hirsutum* plants, which were also most attractive to the wasps. This result was expected as several studies have demonstrated dose-dependent responses of natural enemies (Turlings et al. 1990, 1991; Vaughn et al. 1996). Nonetheless, Hoballah et al., 2002 showed – with maize and cowpea – that not only the quantity of the volatile emissions, but also the quality (i.e. composition or blend) of the volatile profile is important for attraction of *C. marginiventris*, and other studies with the same parasitoid suggest that specific compounds that are released in minor amounts are of key importance for attraction (D’Alessandro et al. 2009; Sobhy et al., 2014). We found that the amount of emitted green leaf volatiles did not differ between the two cotton species, and surmise that GLVs are not likely to be responsible for the observed differences in attracting *C. marginiventris*, even though it is innately attracted to GLVs (Hoballah et al. 2002). The main difference between the emissions of both cotton species was in their monoterpene and sesquiterpene emissions, suggesting that these classes of compounds could be important for the attraction of *C. marginiventris* to cotton plants. Overall, our results indicate that volatile terpenes were more likely to act as cues to attract wasps towards *G. hirsutum* damaged plants than the GLVs.

As this study demonstrates, growing conditions strongly affect the constitutive levels of metabolites that are involved in direct and indirect chemical defences, but remarkably also their levels upon defence induction. This of course has important implications for the biotic interactions that are studied under different experimental conditions, and could explain some of the differences frequently observed between field and laboratory experiments. Based on our results and those of others (Clavijo McCormick 2016), we recommend that researchers bear in mind the potential effects of abiotic laboratory growth conditions on plant responses to herbivory and defence levels, and consider these effects when interpreting results. It seems possible to select the growth conditions that are most appropriate for the specific research questions, and to consider the risk of under/overestimating the effect of induction on defences. Although this study included only two species of cotton, the clear results suggest that other model plant species grown under different conditions will exhibit similar differences. We hope that this study will be of use for the experimental design of future studies on plant defences.

*Conclusions.* To our knowledge this is the first study to specifically test and demonstrate that growth conditions can significantly influence the induction of direct and indirect defence compounds. These differences could lead to biases in the interpretation of the results in studies of plant defences depending on the environmental conditions used; however, it is worth noting that in general the defence patterns were largely similar for the two growth conditions. We further confirm that mechanically damaging the leaves of cotton plants and treating them with caterpillar regurgitant triggers increases in direct (chemical) defences as well as indirect defences (VOC emissions). The analyses also revealed quantitative and qualitative differences between the two cotton species *G. herbaceum* and *G. hirsutum*. Chemical defences were highest in the youngest leaf, and this difference was greatly enhanced following induction, as predicted by the optimal-defence-theory. This study highlights the importance of the conditions under which experimental plants are grown for studies on plant defence induction and defence ontogeny. Our results imply that, so far, the importance of this factor has been greatly underestimated in such studies.

## Electronic Supplementary Material

Below is the link to the electronic supplementary material.

Supplementary Material 1

## Data Availability

The data supporting the findings of this study and associated code can be found at the Zenodo repository 10.5281/zenodo.7713165 (Bustos-Segura et al. 2023).
