# Supplementary material for: Experimental Growth Conditions affect Direct and Indirect Defences in two Cotton Species
Source: J Chem Ecol. 2023 May 9;49(5-6):340–52. doi: 10.1007/s10886-023-01422-5 (PMC10495274; doi:10.1007/s10886-023-01422-5)
Supplement: Supplementary file 1 — Supplementary Material 1 [file 10886_2023_1422_MOESM1_ESM.doc]

SUPPLEMENTARY MATERIALS

**Table S1:** P-values indicating significant effects of the growth condition (greenhouse *versus* phytotron), the cotton species (*G. hirsutum versus G. herbaceum*), the induction status (undamaged *versus* damaged) and their interactions on the amount of VOC released from plants. All variables were analysed with a generalized linear model with a Gamma distribution and a log link. P values were corrected for multiple testing using a false discovery rate correction. Values in bold text indicate P<0.05. Compounds followed by an asterisk were authenticated with commercial standards.

| **Compound(s)** | **Species (S)** | **Condition (C)** | **Induction (I)** | **S*C** | **S*I** | **C*I** |
| --- | --- | --- | --- | --- | --- | --- |
| **GLV** | 1.000 | 1.000 | **0.000** | 1.000 | 1.000 | 0.545 |
| (Z)-3-hexanal * | 1.000 | 1.000 | **0.000** | 1.000 | 1.000 | 1.000 |
| (E)-2-hexenal * | 1.000 | 0.207 | **0.000** | 1.000 | 1.000 | 0.207 |
| (Z)-3-hexenol * | 1.000 | 1.000 | **0.000** | 1.000 | 1.000 | 1.000 |
| (Z)-3-hexanyl-acetate * | 1.000 | 1.000 | **0.000** | 1.000 | 1.000 | 1.000 |
|  |  |  |  |  |  |  |
| **Monoterpenes** | 0.986 | 1.000 | **0.000** | 0.266 | **0.015** | **0.001** |
| α-phellandrene | **0.000** | **0.000** | **0.000** | **0.000** | **0.000** | **0.000** |
| α-pinene * | 0.529 | 1.000 | **0.000** | 0.388 | **0.005** | **0.000** |
| Camphene | 1.000 | 1.000 | **0.000** | 1.000 | **0.001** | **0.000** |
| β-phellandrene | 0.758 | 1.000 | **0.000** | 0.905 | 0.717 | 1.000 |
| β-pinene | 1.000 | 1.000 | **0.000** | 0.550 | **0.008** | **0.008** |
| Myrcene * | 1.000 | **0.000** | **0.000** | 1.000 | 0.297 | **0.002** |
| Limonene * | 1.000 | 1.000 | **0.000** | 0.344 | 0.873 | 0.873 |
| (E)-β-ocimene * | 1.000 | 1.000 | **0.000** | 1.000 | 0.215 | **0.000** |
| Linalool * | 1.000 | **0.014** | **0.009** | 1.000 | 1.000 | 1.000 |
|  |  |  |  |  |  |  |
| **Homoterpenes** | 1.000 | **0.000** | **0.000** | 0.471 | 0.164 | 0.600 |
| DMNT | 1.000 | **0.001** | **0.000** | 0.166 | **0.040** | 1.000 |
| TMTT | 0.693 | **0.029** | 1.000 | 1.000 | 1.000 | 0.473 |
|  |  |  |  |  |  |  |
| **Sesquiterpenes** | **0.028** | **0.001** | **0.000** | 1.000 | 1.000 | 1.000 |
| α-copaene * | 1.000 | **0.001** | **0.001** | 1.000 | 1.000 | **0.001** |
| β-caryophyllene * | **0.013** | **0.000** | **0.000** | 1.000 | 1.000 | 1.000 |
| β-bergamotene | 1.000 | 0.419 | **0.028** | 1.000 | 1.000 | **0.003** |
| α-humulene * | 0.097 | **0.029** | **0.000** | 1.000 | 1.000 | 1.000 |
| β-farnesene * | 1.000 | 0.054 | **0.000** | 1.000 | 1.000 | **0.045** |
| γ-elemene | **0.003** | 0.943 | **0.000** | 0.943 | **0.001** | 0.943 |
| α-farnesene | 0.318 | **0.012** | **0.000** | 1.000 | 1.000 | 1.000 |
|  |  |  |  |  |  |  |
| **Other compouds** |  |  |  |  |  |  |
| Indole * | 0.125 | **0.000** | **0.000** | 0.083 | 0.083 | **0.000** |
| 1-decyne | 1.000 | 0.445 | **0.000** | 1.000 | 1.000 | 1.000 |
| Benzaldehyde * | **0.005** | **0.000** | **0.000** | 0.329 | 1.000 | 1.000 |
| Unknown 1 | **0.006** | **0.005** | **0.000** | 1.000 | 1.000 | 0.191 |
| Unknown 2 (aromatic) | 1.000 | **0.000** | 0.796 | 1.000 | 1.000 | 0.796 |


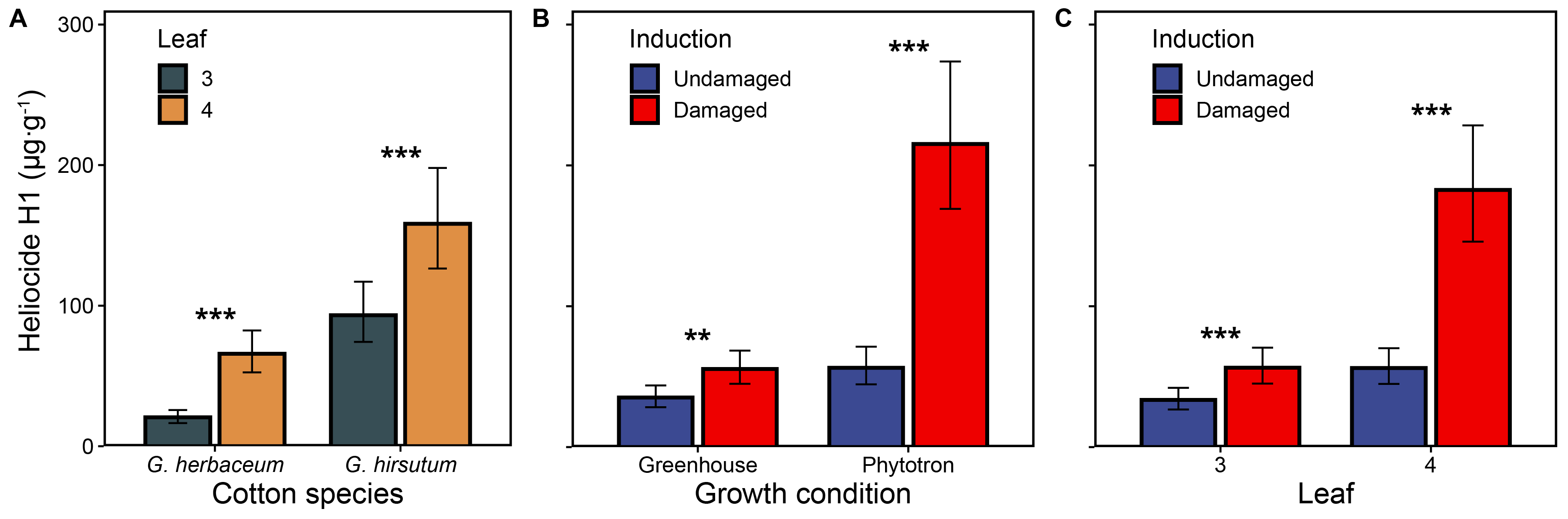


**Figure S1. Effects of species and experimental conditions on heliocide H1 concentrations. A**: cotton species (*G. herbaceum* or *G. hirsutum*) by leaf stage (leaf 3 or leaf 4); **B**: growth condition (greenhouse or phytotron) by induction status (undamaged or damaged); and **C**: leaf stage by induction status. Values for each panel are marginal mean estimates and 95% C.I. from the GLM for focal factors at averaged levels of non-focal factors. Undamaged = plants kept intact as control, damaged = plants induced by mechanical damage and application of caterpillar regurgitant. Probability levels: * P<0.05, ** P<0.01, *** P<0.001.


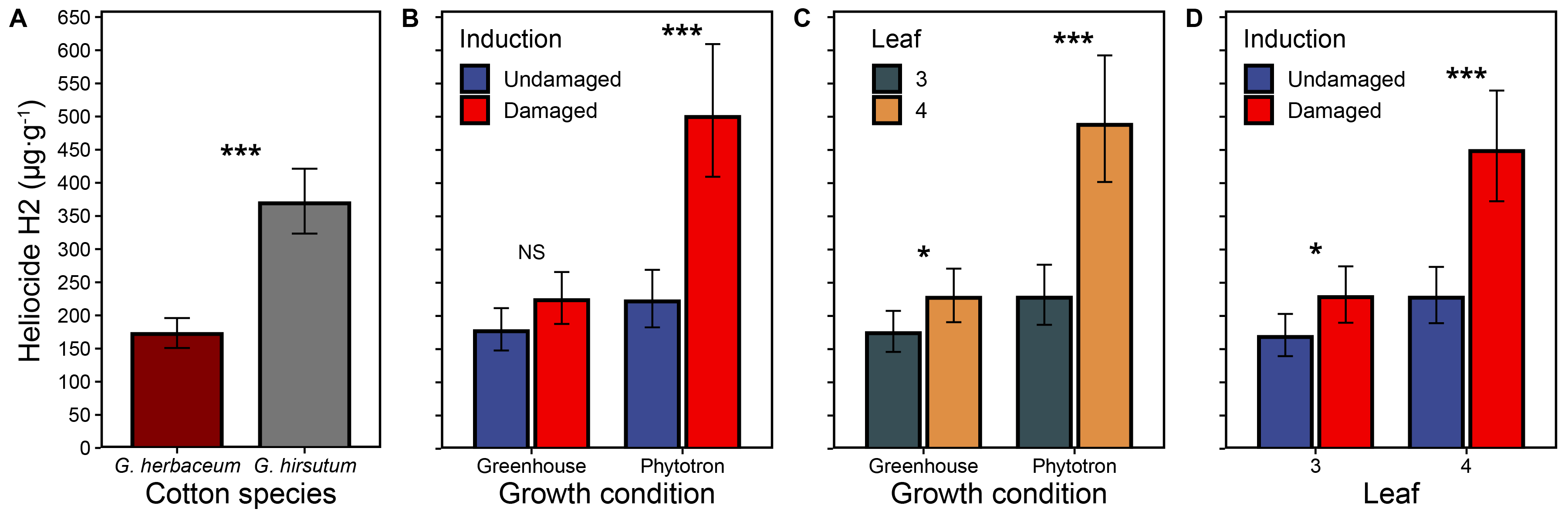


**Figure S2. Effects of species and experimental conditions on heliocide H2 concentrations. A**: cotton species (*G. herbaceum* or *G. hirsutum*); **B**: growth conditions (greenhouse or phytotron) by induction status (undamaged or damaged); **C**: growth conditions by leaf stage (leaf 3 or leaf 4); and **D**: leaf stage by induction status. Values for each panel are marginal mean estimates and 95% C.I. from the GLM for focal factors at averaged levels of non-focal factors. Undamaged = plants kept intact as control, damaged = plants induced by mechanical damage and application of caterpillar regurgitant. Probability levels: * P<0.05, ** P<0.01, *** P<0.001; NS: P>0.05.


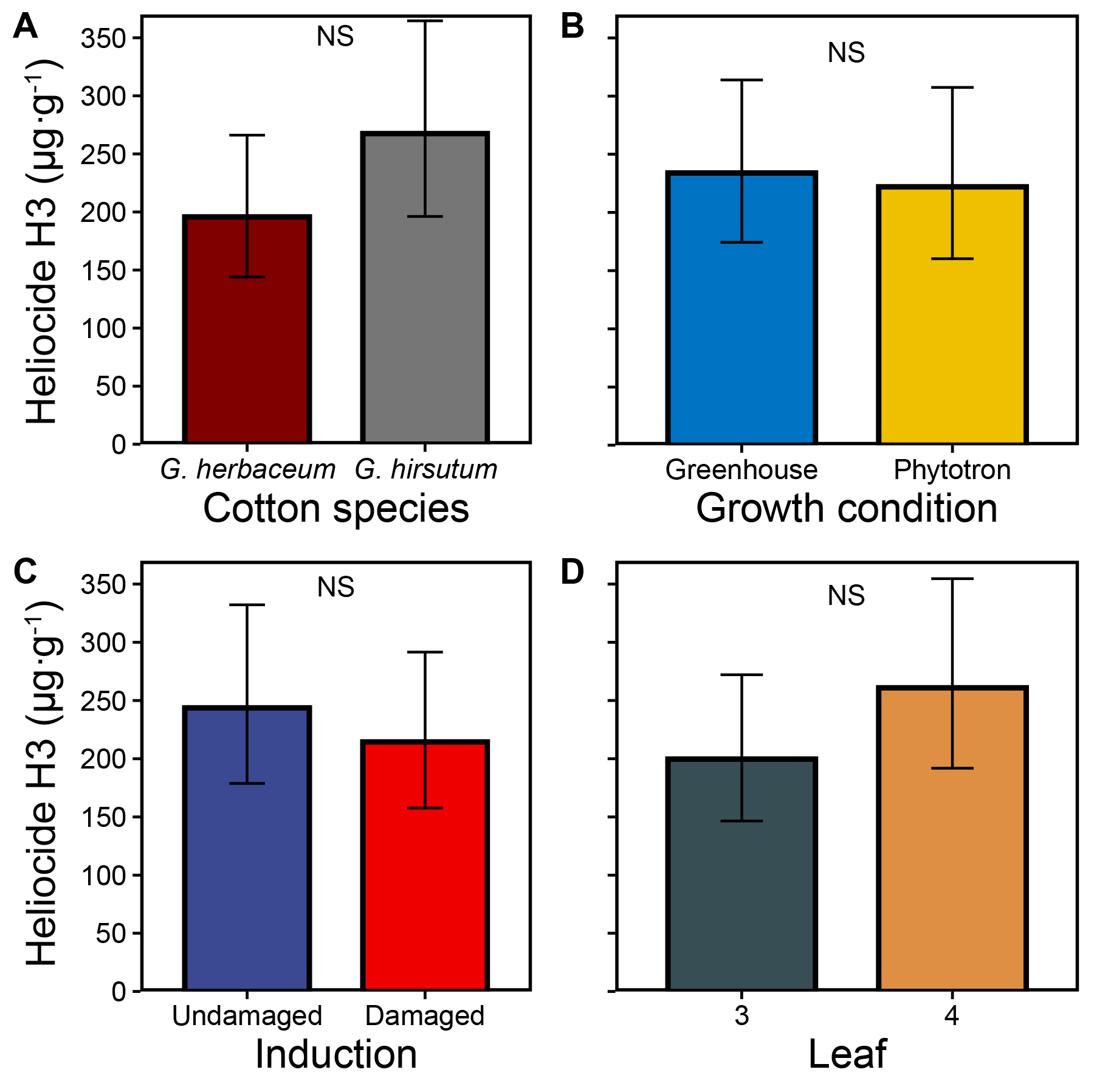


**Figure S3. Effects of species and experimental conditions on heliocide H3 concentrations. A**: cotton species (*G. herbaceum* or *G. hirsutum*); **B**: growth conditions (greenhouse or phytotron); **C**: by induction status (undamaged or damaged); and **D**: leaf stage (leaf 3 or leaf 4); and **D**: leaf stage by induction status. Values for each panel are marginal mean estimates and 95% C.I. from the GLM for focal factors at averaged levels of non-focal factors. Undamaged = plants kept intact as control, damaged = plants induced by mechanical damage and application of caterpillar regurgitant. Probability levels: NS: P>0.05.

**
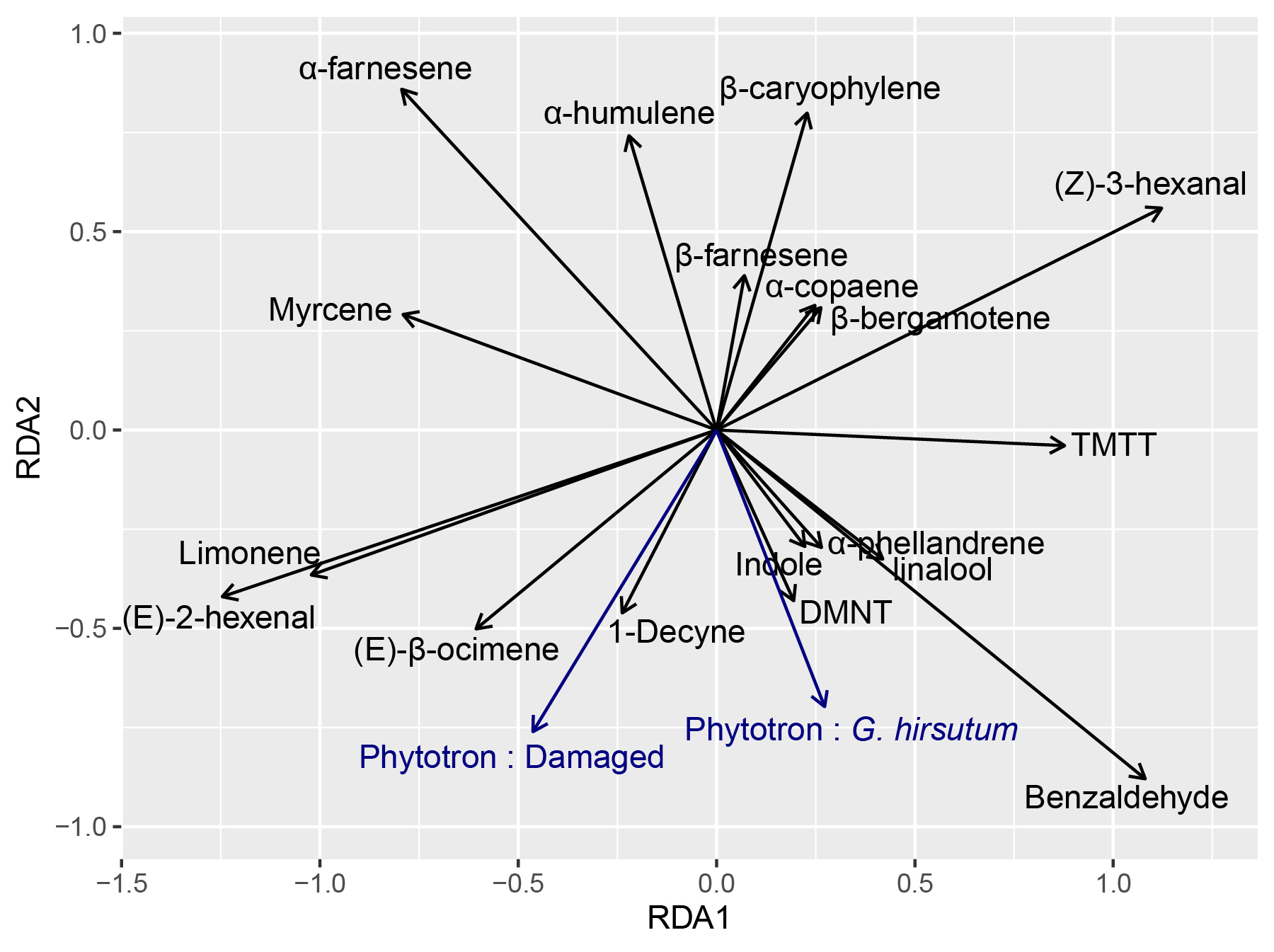
**

**Figure S4. Redundancy analysis biplot of individual VOCs by plant treatments.** Black vectors show the main individual VOCs, with name labels for the most important VOCs. Blue arrows show the direction of the interaction between factors.
